# Effects of Curing Conditions on Pore Structure of Ultra-High-Strength Shotcrete (UHSSC) Based on X-ray Computed Tomography

**DOI:** 10.3390/ma17164094

**Published:** 2024-08-18

**Authors:** Shijie Xiao, Jianyu Yang, Zelin Liu, Weijun Yang, Jiangang He

**Affiliations:** 1 College of Civil Engineering, Changsha University of Science & Technology, Changsha 410076, China; xsj201607@163.com (S.X.); lzl597489330@163.com (Z.L.); yyyaozhijian@163.com (W.Y.); 2 School of Civil Engineering and Architecture, Jishou University, Zhangjiajie 427000, China; 3 Hunan Renjian Baogu High-Tech Development Co., Ltd., Changsha 410005, China; 13307315359@163.com

**Keywords:** UHSSC, curing condition, pore structure, compressive strength, grey theory, X-CT

## Abstract

Shotcrete is widely used in mine and civil engineering as supporting structure. A new type of ultra-high-strength shotcrete (UHSSC) with viscosity-enhancing agent was taken as the research object in this paper. A microstructure model of UHSSC under different curing conditions (standard curing, natural curing and film curing) was reconstructed using X-ray computed tomography (X-CT). The grey theory was used to analyze the correlation between pore characteristics and strength of UHSSC. The results showed that the porosity and the pore size of UHSSC were significantly reduced, the compressive strength was obviously improved by the new spraying process. The effects of curing conditions on the pore characteristics and compressive strength of UHSSC were obvious. Under natural curing, the hydration degree was the highest, the maximum pore size was the smallest, and the compressive strength was the highest, reaching 95.8 MPa, but the porosity was the highest. The curing condition had a certain influence on the sphericity distribution of UHSSC pores. Under film curing, the proportion of special-shaped pores (S < 0.4) was the largest and compressive strength was the smallest. There was a good correlation between pore characteristic parameters and the compressive strength of UHSSC under different curing conditions. In particular, the large pore size (D ≥ 5000 µm) and special-shaped pores (S < 0.4) had obvious effects on the strength of UHSSC, and the grey correlation coefficients were 0.8539 and 0.8080, respectively. Additionally, the pore direction of UHSSC had obvious directionality, and the anisotropy of UHSSC may be more prominent than poured specimen. The results will lay a foundation for the study of its mechanical properties and durability.

## 1. Introduction

Shotcrete is conveyed under pressure through a pneumatic hose or pipe and used to the target surface by spraying it at high speed [1]. It has the advantages of simple process, efficient construction, good bonding performance, economic cost, and strong adaptability and has been widely applied to underground engineering, tunnel engineering, slope support, water conservancy engineering, restoration and reinforcement engineering and other fields [2,3,4,5,6,7]. Shotcrete is a kind of 3D printing concrete molding technology and can be directly formed based on the spray surface, so it is a type of additive manufacturing (AM) technology [8,9,10]. Therefore, it has attracted the attention of many scholars.

In order to shorten the setting time of concrete, accelerate the hardening process of the cement, and increase the thickness of a spray, accelerators are added to shotcrete in the traditional construction process [11,12]. Most of the accelerators shorten the concrete’s setting time and improve the early strength but cause long-term strength loss and shrinkage [13,14,15,16] and even cause alkali aggregate reaction [17,18,19]. The main reason is that the accelerators significantly affect the hydration process of cement, changing the hydration degree of different cement minerals and morphology and the distribution of hydration products [13].Therefore, the addition of accelerators inevitably changes the microstructure of cement-based materials and increases porosity [20,21,22,23,24]. The above research results showed that accelerators had an important influence on the pore characteristics and properties of shotcrete.

**Figure 1:**
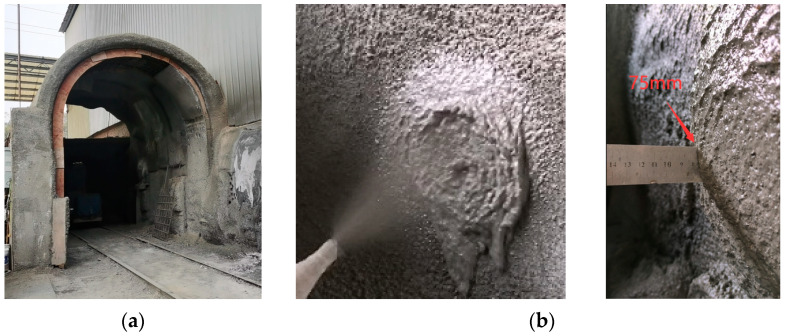
Site spray trial. (**a**) Trial site. (**b**) One-spray thickness 75 mm.

In order to reduce the porosity of shotcrete, the research team has developed a new technology of wet-mixed shotcrete. The link of adding liquid accelerator at the nozzle position in wet-mix shotcrete was creatively cancelled by adding viscosity-enhancing agent in premix [5], which contained all materials except water, such as cement, mineral admixtures, aggregates, fibers, etc. Through on-site spraying, the construction performance of UHSSC was verified, and the sprayed thickness (side wall) could be up to 75 mm, as shown in Figure 1. In order to better understand the influence of the viscosity-enhancing agent on the macroscopic properties of UHSSC, it is necessary to study pore characteristics.

Various experimental methods have been applied to study the microstructure of cement-based materials. In general, these methods can be divided into indirect and direct methods [25]. In the indirect methods, the pore characteristics are obtained by using external stimulation to detect the reaction of the tested material, such as mercury intrusion porosimetry (MIP), gas adsorption methods, and thermoporometry. Among them, MIP is the most widely used, but the pore structure may be damaged by drying pretreatment and high intrusion pressure in the test process [26,27,28]. In addition, the obtained results are indirect and cannot be directly related to the actual microstructure. In contrast, direct methods, including X-ray computed tomography (X-CT), back-scattered electron imaging (BSEI) and scanning electron microscopy (SEM), use physical images of materials to reveal the size, morphology and spatial distribution of pores. BSEI can only observe the polished two-dimensional section, and it is difficult to reveal the three-dimensional pore spatial distribution characteristics [29]. The samples tested using SEM are too small to be representative of the cement matrix, and it is also destructive to obtain the desired results [30]. X-CT can directly measure the three-dimensional characteristics and distribution of pores in solid materials, which is a non-invasive and non-destructive measurement method. At present, it has been widely used to characterize the microporous structure of concrete, such as ultra-ultra-high concrete [31], pervious concrete [32], foam concrete [33], and asphalt concrete [34]. It can also be applied to study the pore characteristics of UHSSC.

Due to the influence of external environment and internal hydration, the pore structure of concrete will be changed by hydration products, and these changes will directly affect the mechanical properties and durability of concrete [35,36,37,38,39]. At present, most of the research on pore characteristics of concrete is carried out under standard curing conditions, but the curing conditions of components or structures in actual engineering are difficult to meet the requirements of laboratories. The pore characteristics of UHSSC are also affected by curing conditions. However, there are also few studies on the pore characteristics of shotcrete and its relationship with mechanical properties. Therefore, it is necessary to study the pore structure characteristics of UHSSC under different curing conditions, such as porosity, pore diameter, sphericity, etc.

In the paper, a 3D microstructural model of UHSSC was reconstructed under different curing conditions via X-CT, and the pore structure characteristics were obtained using the image processing software. Based on the grey theory, the correlation between the pore characteristic parameters and the compressive strength of UHSSC under different curing conditions was analyzed. The research results will lay a foundation for the study of its mechanical properties and durability.

## 2. Materials and Methods

### 2.1. Materials and Mix of UHSSC

A P•II52.5 Portland cement complying with the Chinese standard GB175-2007 [40] was used as the main cementitious materials in this study. The mineral admixture was fly ash (FA) and silica fume (SF). Artificial sand (AS) with a maximum particle size of 4.75 mm was used as fine aggregate, and there was no coarse aggregate. The particle size was divided according to the Chinese Industrial standard JG/T 568-2019 [41]. A polycarboxylic-acid-based super-plasticizer which is known to be applied to high strength concrete was used. In order to improve the bonding strength during spraying, a viscosity-enhancing agent (VE) was added [5]. To prevent early shrinkage cracking, polypropylene fiber (PP) was also considered. The mix proportion was shown in Table 1.

### 2.2. Preparation of Concrete Specimens

The specimens of UHSSC used in this research were prepared conforming to the Chinese standard JGJ/T 372-2016 [1]. Premix of the UHSSC and ordinary tap water were combined in a concrete mixer to prepare the fresh concrete. Then, it was pumped from the spray pipe to the nozzle by the wet-spray machine and sprayed into 3 steel formworks. The size of the formwork was 450 mm × 450 mm × 120 mm. The angle between the formwork and the ground was about 80°. The distance between the nozzle and the sprayed surface ranged from 0.6 m to 1.0 m. After 1 day of sprinkling water curing, the slabs were demolded from the formworks and moved to the room according to the curing conditions in Table 2 for 28 days. Natural curing represented the curing environment of components in practical engineering. Film curing meant that water could neither be obtained from the external environment nor lost to the external environment. Lastly, the UHSSC slabs were cut into cubes of 100×100×100 mm^3^ by a cutting machine. At the same time, the poured specimens with the same mix proportion were prepared, the curing conditions were standard curing, and the specimens were numbered as MC.

### 2.3. CT Analysis Methods

#### 2.3.1. Principles of CT Image

CT is a three-dimensional imaging technology, which is completely different from general radiation imaging. CT equipment is mainly composed of radioactive sources, detectors and computer systems. Since there are two types of regions in UHSSC, cement base and pore, rays will have different degrees of attenuation when penetrating different regions. The detector receives and generates an electrical signal containing the ray attenuation information, and then transmits it to the computer system through the data collector, so that the real image of a section of the measured object can be obtained. Finally, these two-dimensional images are assembled together to reconstruct three-dimensional images. The process avoids the interference and influence of the rest of the parts, and the image quality is high, which can clearly and accurately show the internal structural relationship of the measured parts.

#### 2.3.2. CT Image Processing Method

In this study, X-ray CT system produced by the China Institute of Engineering Physics was used to obtain the CT image of concrete. The voltage of the X-ray source was 10–240 kV, the minimum focal spot size was 1 µm, the maximum tube power of the instrument was 320 W, the tube current was 0.01–3.0 mA, the effective area of the imaging window was 409.6 mm × 409.6 mm, and the number of pixels was 2048 × 2048. The spatial resolution of the scanned image was 86.8 µm/voxel. Therefore, the pore was too small to be identified, so the porosity value was actually less than the absolute porosity of the concrete.

**Figure 2:**
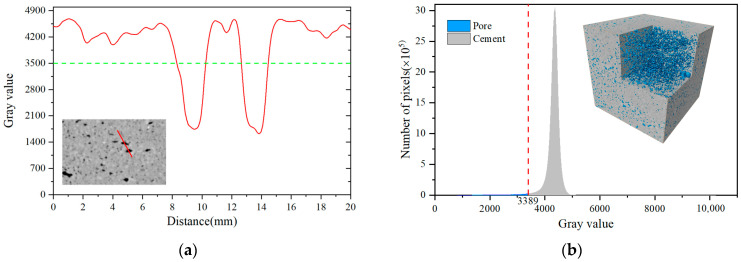
Threshold segmentation. (**a**) Grayscale distribution. (**b**) Greyscale histogram.

In order to analyze the range of grayscale values between cement matrix and pores in the MC specimen, a two-dimensional slice image was randomly selected from the CT im-ages of MC, and then a line segment (the red line in the image of Figure 2a) passing through the cement matrix and the pore was drawn arbitrarily. The gray value distribution curve along the line segment was obtained by using Avizo (Thermo Fisher Scientific, Waltham, MA, USA), as shown in Figure 2a. Avizo can perform image processing and threshold segmentation of different components and then carry out 3D reconstruction and analyze the pore characteristics of the test block. From the gray value curve, it can be seen that the preliminary estimate for the gray threshold of cement matrix and pore was about 3500. Then, the gray value of the whole MC was extracted and the gray distribution histogram was made, as shown in Figure 2b. Because the UHSSC in this paper was a two-phase material (cement matrix and pores), the curve was a typical single-peak curve. The Otsu method [42], based on the maximum variance theory, was used for threshold segmentation, and the threshold obtained was 3389, which is close to 3500. The results show that this method can obtain a reasonable gray threshold value of cement matrix and pores. The other UHSSC specimens were threshold segmented by referring to the same method.

**Figure 3:**
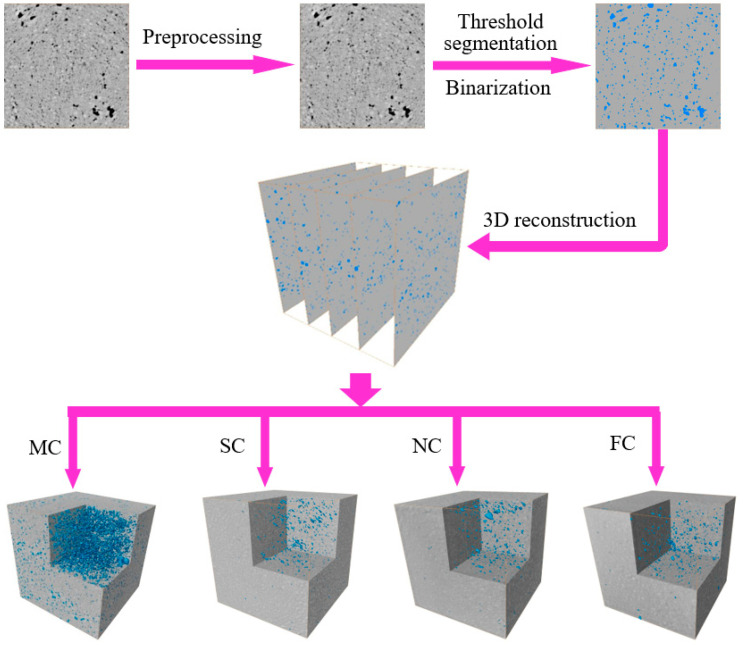
Three-dimensional reconstruction model.

In order to study the 3D pore structure characteristics of UHSSC, the original images were preprocessed (image adjustment, smoothing, sharpening, contrast enhancement and noise processing) to improve the image quality. Then the pores were extracted from the solid skeleton by binarizing the image, and the 3D reconstruction model was carried out to quantitatively analyze the pore structure. Figure 3 showed the 3D reconstruction process.

## 3. Results and Discussion

### 3.1. Porosity

From the 3D reconstruction model, it was evident that the porosity of MC (poured specimen) was much higher than that of the sprayed specimens, with a porosity of 2.634%. The porosities of the three sprayed specimens were close—SC 0.212%, NC 0.260%, and FC 0.189%. The results showed that the new spray process made the concrete denser, which was beneficial for resisting external erosion and improving durability. Among the UHSSC specimens, the porosity of natural curing was the largest and the porosity of film curing was the smallest. This phenomenon was consistent with the results of Luo et al. [35].

**Figure 4:**
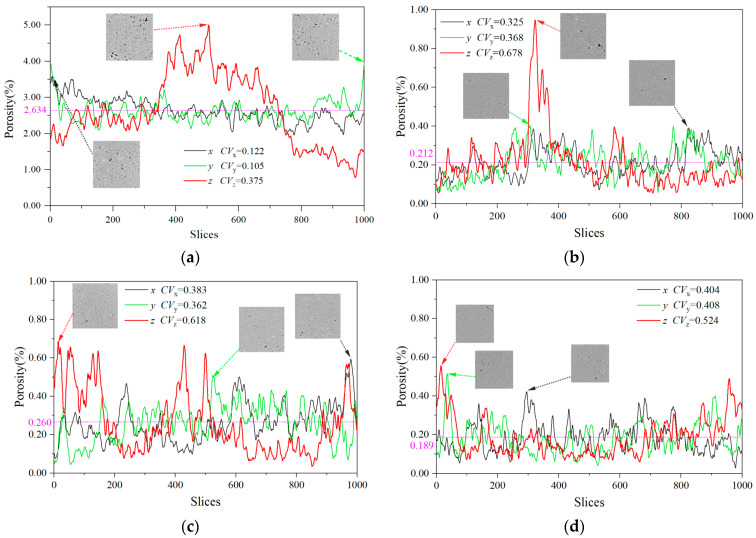
Analysis of 2D porosity. (**a**) MC. (**b**) SC. (**c**) NC. (**d**) FC.

In order to analyze the uniformity of pore distribution, statistical analysis was con-ducted on the plane porosity in the x, y, and z directions of each test block, with the z direction being the spraying or pouring direction. The analysis results are shown in Figure 4. The plane porosity curves showed that the porosity was random. Rules of variation were similar, and the coefficient variation of porosity was close in the x and y directions of each test block. Compared to the UHSSC, the curves of MC were obviously smoother, and the variation coefficient of was also significantly lower. For the same test block, the variation coefficient in z direction was the largest, with MC 0.375 and UHSSC both exceeding 0.5. The results indicated that the pore distribution was directional, and the z direction was different from the x and y directions. In particular, the differences of UHSSC were more obvious.

### 3.2. Pore Size Distribution

Since most of the pores were irregular, it was more appropriate to represent the pore size characteristics as a sphere of the same area [43]. The diameter of the sphere was the equivalent diameter of this pore. The calculation formula of equivalent diameter is as follows:

(1) $$D=\sqrt{A/\pi }$$

where *A* is pore surface area.

**Figure 5:**
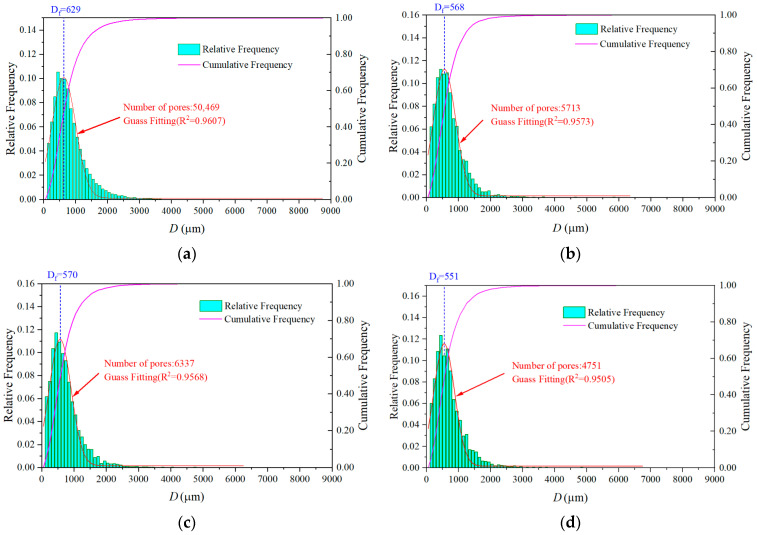
Pore size distribution. (**a**) MC. (**b**) SC. (**c**) NC. (**d**) FC.

The statistical results of pore diameter are shown in Figure 5. It can be seen that the pore size distribution accorded with the normal distribution well, and this indicated that the process of pore size changing from small to large was smooth and gradual without abrupt change. However, the three indicators of pore quantity, average pore size (Gauss fitting), and maximum pore size of MC were significantly different from those of UHSSC. The number of MC pores was 50,469, while the numbers of UHSSC pores were approximately one-tenth of the MC. The average pore size of MC was 629 µm, while the average pore size of UHSSC was around 560 µm. The maximum pore size of MC was 8719 µm, while the maximum pore sizes of the UHSSC specimens were 6303 µm (SC), 6221 µm (NC), and 6664 µm (FC), respectively. The results showed that the spray process significantly reduced the number and diameter of pores. In the UHSSC specimens, the maximum pore size of the film curing was the largest, but the average pore size was the smallest and the number of pores was the least. The maximum pore size of the natural curing was the smallest, but the average pore size was the largest and the number of pores was the highest. The reason for this phenomenon may be that the degree of hydration for natural curing was the highest, while film curing was the lowest. In the hydration process, the hydration products in the concrete overflowed, which would generate pores and increase the number of pores. At the same time, the hydration products filled the original pores and reduced the pore size of the original pores [35]. This also caused the porosity of film curing to be the lowest, while natural curing was the highest.

### 3.3. Pore Morphology Analysis

Previous studies have shown that the mechanical properties of concrete are greatly affected by internal pore defects [44,45,46]. Similarly, the pore shape will affect the performance of shotcrete to a certain extent. In sedimentology, sphericity is used to describe the shape of particles and reveals the transportation and depositional mechanisms [47,48]. For pore morphology analysis, sphericity also can be used to analysis by considering the pore resembling a particle. Pore sphericity is defined as the ratio of the sphere surface area with the same volume as the pore to the pore surface area of the pore. The calculation formula of pore sphericity is as follows [49]:

(2) $$S=\frac{\;\pi^{\frac{1}{3}}{(6V)}^{\frac{2}{3}}}{A}$$

where *V* is pore volume and *A* is pore surface area.

**Figure 6:**
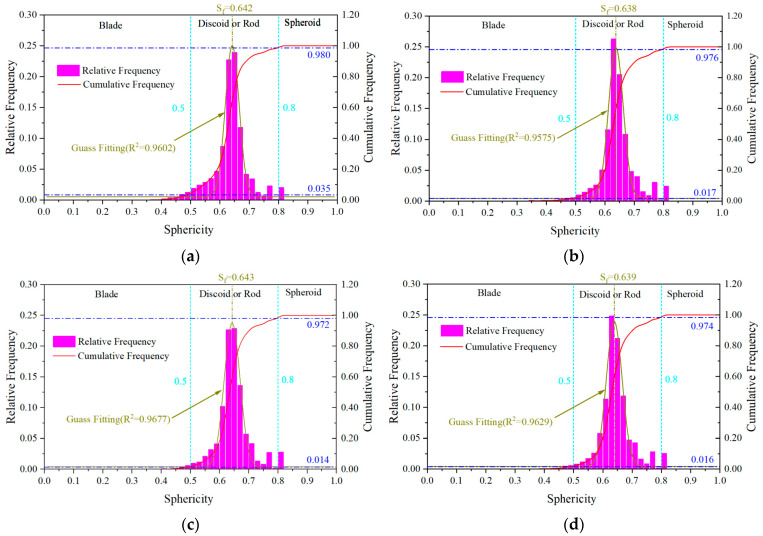
Sphericity distribution. (**a**) MC. (**b**) SC. (**c**) NC. (**d**) FC.

Referring to the shape classification of sediment particles by Zingg [50], pore shapes can be divided into three categories: blade (S ≤ 0.5), discoid or rod (0.5 < S < 0.8) and spherical (S ≥ 0.8). The influence of the blade shapes pores on the mechanical properties is the most prominent [51]. Figure 6 showed the statistical analysis results of sphericity. The frequency distribution of sphericity was similar and showed a single peak distribution, and the frequency in the interval of 0.5–0.8 exceeded 94%, indicating that most of the pores exist as discoids or rods. Compared to pouring, the proportion of spherical pores was slightly increased and the proportion of blade pores was reduced by spraying for UHSSC. It was likely that the spray process changed the pore shape distribution.

**Figure 7:**
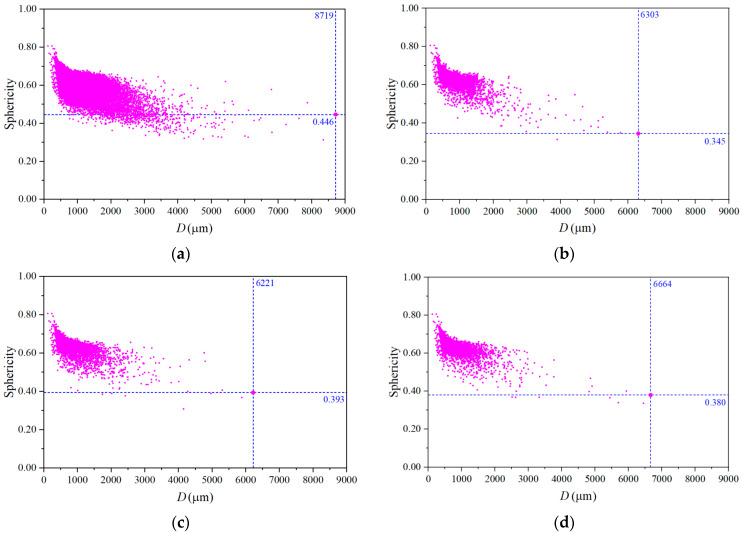
Sphericity–pore diameter scatter diagram. (**a**) MC. (**b**) SC. (**c**) NC. (**d**) FC.

The sphericity–pore diameter scatter diagram was drawn, as shown in Figure 7. It can be seen that the pore sphericity distribution was related to the pore size, and with the increase of diameter, the sphericity tended to decrease. The sphericity corresponding to the maximum pore size of MC was 0.446, while the sphericity corresponding to the maxi-mum pore size of UHSSC specimens was less than 0.4. These indicated that while the pore size decreased during the spray process, the sphericity of the pores may be reduced. In particular, the sphericity of the macropores varied greatly, making the shape of the macropores more irregular.

### 3.4. Anisotropy Analysis

Anisotropy is a very important feature of materials, which can reflect the location and failure trend of material weaknesses or cracks [49]. From the analysis results of plane porosity in Section 3.1, it was found that the pore distribution was directional, and the z direction was different from the x and y directions for the same test block. Especially, the differences of UHSSC were more obvious. In order to further study the anisotropy of material properties, the method used in CT image analysis to characterize the direction of fiber path in biology [52] was applied to calculate the direction of pore and analyze the anisotropy of shotcrete.

**Figure 8:**
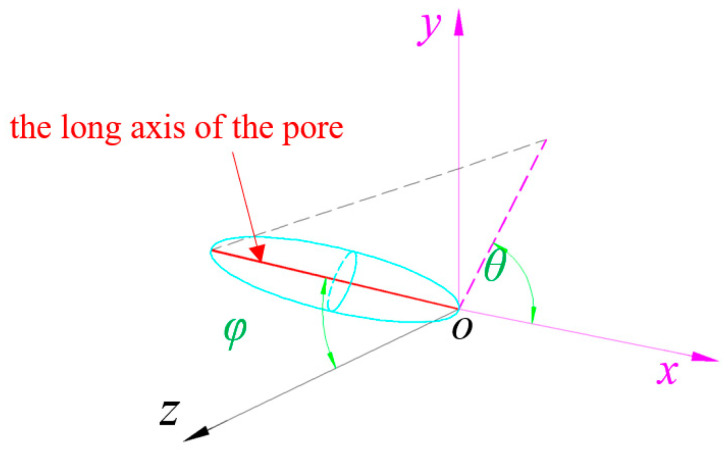
Schematic diagram of pore orientation.

**Figure 9:**
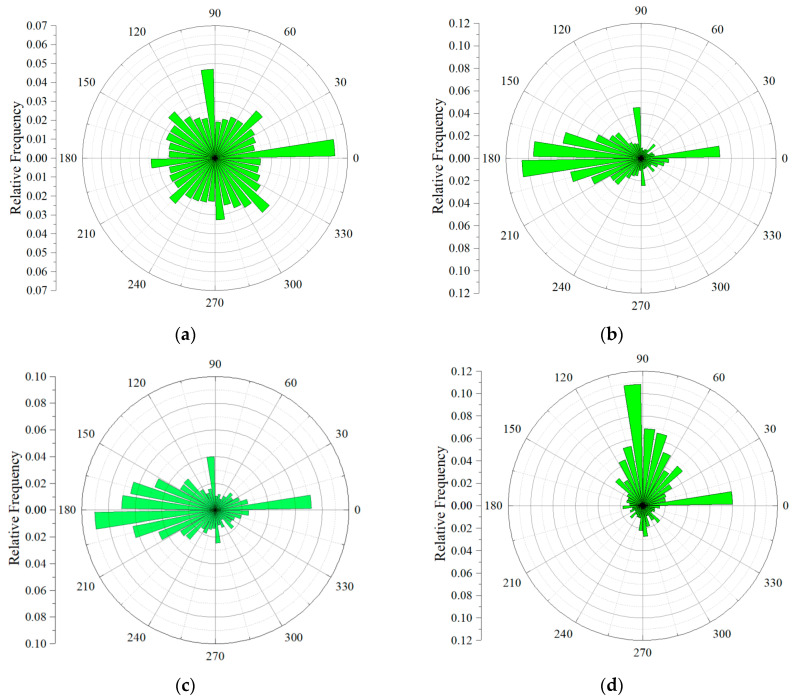
Rose map of *θ*. (**a**) MC. (**b**) SC. (**c**) NC. (**d**) FC.

**Figure 10:**
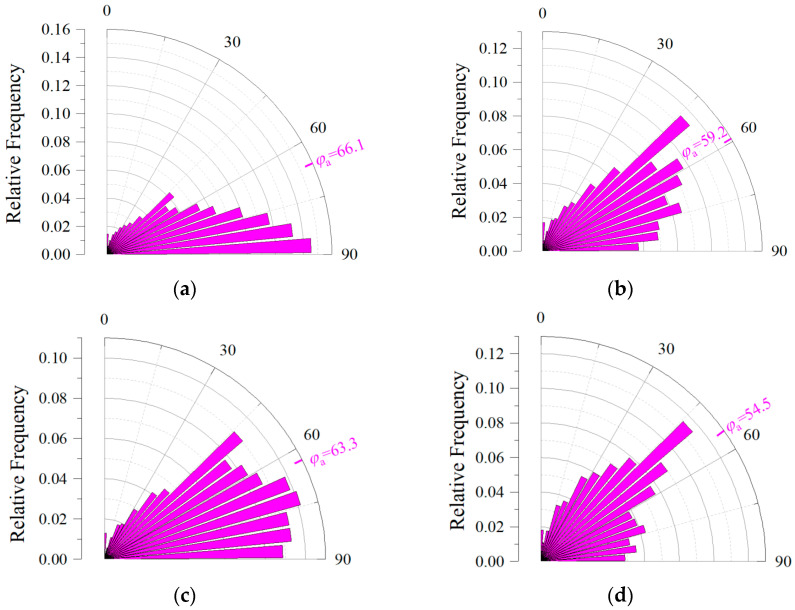
Statistical chart of the frequency of *φ*. (**a**) MC. (**b**) SC. (**c**) NC. (**d**) FC.

The pore direction was defined as the direction of the long axis of the pore, which is the eigenvector of the largest eigenvalue of the covariance matrix [49]. In this paper, Avizo 2020.1 software was used to calculate the pore direction, and schematic diagram of pore orientation was shown in Figure 8 (the z direction was the spraying direction or the pouring direction). The angle between the projection of the long axis of the pore in the x–y plane and *X*-axis was *θ* (0–360). The angle between the long axis of the pore and the *Z*-axis was *φ* (0–90), where 0 indicated horizontal and 90 indicated vertical. The statistics details of *θ* are shown in a rose map in Figure 9. It can be found that the overall distribution of pore direction projection of MC in the x–y plane was relatively uniform, but the projection of UHSSC in the x–y plane had an obvious tendency to concentrate in a certain direction (near the *X*-axis or *Y*-axis). Figure 10 showed the statistical chart of the frequency of *φ*. The average *φ* of MC was 66.1°, while the average *φ* of UHSSC specimens were 59.2° (SC), 63.3° (NC), and 54.5° (FC), respectively. The results illustrated that the distribution of pore direction was obviously changed by the spraying process, and the *θ* was mainly distributed in a certain range. Therefore, the anisotropy of the UHSSC was likely to be more prominent than the pouring specimens.

### 3.5. Analysis of the Influence of Pore Parameters on Compressive Strength

Compressive strength testing was carried out according to the Chinese standard JGJ/T 372 2016 [1]. The compressive strengths of the four groups of test blocks are shown in Table 3. Compared to MC, the compressive strength of UHSSC had been significantly improved. However, under different curing conditions, there were obvious differences in the increase range of compressive strength, and the natural curing had the largest increase, reaching 95.8 MPa, an increasing of 32.9%. Previous research showed that under different curing conditions, the porosity, size, and shape of the pores of UHSSC were different. It was difficult to establish a direct relationship between each single characteristic parameter and the compressive strength. Therefore, in order to comprehensively analyze the influence of the pore characteristic parameters of UHSSC on its mechanical properties, the grey correlation theory was used to evaluate the correlation degree between them in this paper. The compressive strength was set as the reference sequence, and the relevant parameters of pore characteristics were set as the comparison sequence. According to literature [53,54,55], the grey correlation degree between pore characteristic parameters and compressive strength was calculated, and the calculation results are shown in Table 4.

As can be seen from Table 4, under different curing conditions, the grey correlation coefficient between the pore characteristic parameters and the compressive strength of UHSSC was all above 0.65. This also showed that there was a close relationship between the pore characteristics and the strength of shotcrete. The average sphericity (S_f_) had the closest relationship with the compressive strength, and the grey correlation coefficient reached 0.7827. The second was the average size (D_f_), and the grey correlation coefficient reached 0.7472. Under different curing conditions, the effect of porosity and maximum pore size (D_max_) on the compressive strength of UHSSC was relatively weak. In particular, as the porosity increased, the compressive strength increased. This showed that the influence of pore characteristic parameters on concrete strength was very complex. Grey theory was used to further analyze the influence of sphericity distribution and pore size distribution on compressive strength, and the calculated results are shown in Table 5 and Table 6.

It can be seen from Table 5 that in the distribution interval of sphericity of 0–0.4, the grey correlation coefficient was the largest, reaching 0.8080. It showed that the smaller the pore sphericity was, the more complex the pore morphology was, and the more unfavorable the compressive strength was. When the sphericity was in the range of 0–0.4, the proportion of natural curing pores was the smallest and the strength was the largest. However, the proportion of pores in the film curing was the largest, and the strength was the smallest. Table 6 shows that macropores with pore size greater than 5000 μm had the highest correlation with the compressive strength of UHSSC, reaching 0.8539. The above results show that although the porosity of natural curing was the largest, the volume proportion of complex pores (S < 0.4) and macropores (D ≥ 5000 μm) was relatively the lowest, and the compressive strength was the highest. The reason for this phenomenon was that the hydration speed of natural curing was the fastest, and the amount of hydration products spilled by concrete was the largest, which increased the porosity. However, the overflowing hydration products would fill part of the pores, reduce the diameter of the macropores, and increase the sphericity of the macropores, which directly reduced the stress concentration effect around the macropores and improved the compressive strength.

## 4. Conclusions

UHSSC has been widely concerned because of its excellent mechanical properties and durability. However, there are few studies on UHSSC, especially on the pore characteristics of UHSSC under different curing conditions. However, the pore characteristics of UHSSC have an important impact on its strength and durability. Around this problem, a microstructure model of UHSSC under different curing conditions was reconstructed using X-CT, pore characteristics were analyzed quantitatively, and the grey correlation theory was used to analyze the correlation between pore characteristics and compressive strength of UHSSC. The following conclusions can be drawn:

(1) The new spray process significantly reduced the porosity and pore size, so the concrete became denser and the compressive strength was significantly improved. The curing conditions affected the hydration and porosity of UHSSC. Under natural curing conditions, the hydration degree was the highest, the maximum pore size was the smallest, and the compressive strength was the highest, reaching 95.8 MPa, but the porosity was the highest.
(2) The sphericity of pores was closely related to pore size. The sphericity tended to decrease as the pore size increased. The new spray process would reduce the sphericity of the large pores. The curing condition had a certain influence on the sphericity distribution of UHSSC pores. Under the film curing, the proportion of special-shaped pores (S < 0.4) was the largest, and its compressive strength was the smallest.
(3) There was a good correlation between pore characteristic parameters and the compressive strength of UHSSC under different curing conditions. In particular, the large pore size (D ≥ 5000 µm) and special-shaped pores (S < 0.4) had obvious effects on the strength of UHSSC, and the grey correlation coefficients were 0.8539 and 0.8080, respectively.
(4) The pores of UHSSC were obviously directional, the parameter *θ* was concentrated in a certain direction, and the parameter *φ* was significantly reduced compared with the poured specimen. In addition, there were differences in the parameter *φ* of UHSSC under different curing conditions. This indicated that the anisotropy of UHSSC may be more prominent than that of the poured specimen and was affected by the curing conditions at the same time. Further research is needed on this issue.

## Figures and Tables

**Table 1:** Mix proportion of ultra-high-strength shotcrete (UHSSC).

Water	Cement	SF	FA	AS	Superplasticizer	PP	VE
0.295	1.0	0.128	0.103	1.282	0.005	0.1%	0.077

Notes: PP was the volume ratio, and the others were the weight relative to the cement.

**Table 2:** Curing condition.

No.	Forming Process	Curing Regime	Curing Conditions
SC	Spraying	Standard curing	In curing room (20 ± 2 °C and relative humidity ≥ 95%)
NC		Natural curing	Indoors, continue sprinkling water curing for 13 days and then placed for 14 days
FC		Film curing	Indoors, samples were sealed with plastic film to prevent moisture evaporation
MC	Pouring	Standard curing	In curing room (20 ± 2 °C and relative humidity ≥ 95%)

**Table 3:** Compressive strength and pore characteristics.

Parameter		MC	SC	NC	FC
Compressive strength/MPa	A	74.9	88.7	100.7	80.9
	B	77.3	93.5	96.9	83.2
	C	64.1	92.2	89.9	90.4
	Average	72.1	91.5	95.8	84.8
Porosity/%		2.634	0.212	0.260	0.189
D_max_/µm		8719	6303	6221	6664
D_f_/µm		629	568	570	551
S_f_		0.642	0.638	0.643	0.639

Notes: A, B, C were the labels for 3 specimens of each type (MC, SC, NC, FC).

**Table 4:** Grey correlation between pore characteristic parameters and compressive strength of UHSSC.

Characteristic Parameter	Porosity	D_max_	D_f_	S_f_
Correlation	0.6837	0.6689	0.7472	0.7827

**Table 5:** Grey correlation between pore sphericity distribution and compressive strength of UHSSC.

Pore Sphericity Distribution	Proportion of Pores/% by Volume			Correlation
	SC	NC	FC	
0–0.4	0.018732	0.013535	0.018917	0.8080
0.4–0.5	0.037657	0.027366	0.025949	0.6569
0.5–0.6	0.068311	0.097745	0.060358	0.6467
0.6–0.7	0.086530	0.120322	0.083490	0.7021
0.7–0.8	0.000501	0.000549	0.000439	0.7311
0.8–1	0.000034	0.000045	0.000031	0.6826

**Table 6:** Grey correlation between pore size distribution and compressive strength of UHSSC.

Pore Sphericity Distribution/µm	Proportion of Pores/% by Volume			Correlation
	SC	NC	FC	
≥5000	0.0180	0.0112	0.0193	0.8539
5000 > d ≥ 4000	0.0148	0.0186	0.0077	0.6864
4000 > d ≥ 3000	0.0153	0.0188	0.0095	0.7105
3000 > d ≥ 2000	0.0303	0.0565	0.0391	0.6636
2000 > d ≥ 1000	0.0887	0.1054	0.0779	0.7930
1000 > d ≥ 0	0.0447	0.0490	0.0352	0.7935

## Data Availability

The data presented in this study are available on request from the corresponding author. The data are not publicly available due to privacy.
